# Genetic, physiological and comparative genomic studies of hypertension and insulin resistance in the spontaneously hypertensive rat

**DOI:** 10.1242/dmm.026716

**Published:** 2017-03-01

**Authors:** Philip M. Coan, Oliver Hummel, Ana Garcia Diaz, Marjorie Barrier, Neza Alfazema, Penny J. Norsworthy, Michal Pravenec, Enrico Petretto, Norbert Hübner, Timothy J. Aitman

**Affiliations:** 1 Centre for Genomic and Experimental Medicine & Centre for Cardiovascular Science, Queen's Medical Research Institute, University of Edinburgh, Edinburgh EH4 2XU, UK; 2 Cardiovascular and Metabolic Sciences, Max-Delbrück-Center for Molecular Medicine (MDC), 13125 Berlin, Germany; 3 Department of Medicine, Imperial College London, London SW7 2AZ, UK; 4 MRC Clinical Sciences Centre, Imperial College London, London W12 0NN, UK; 5 Department of Model Diseases, Institute of Physiology, Czech Academy of Sciences, 142 20 Prague, Czech Republic; 6 Duke-NUS Medical School, Singapore 169857, Republic of Singapore; 7 DZHK (German Centre for Cardiovascular Research), partner site, 13316 Berlin, Germany; 8 Charité-Universitätsmedizin, 10117 Berlin, Germany

**Keywords:** Rat, Congenic, Genomic, Hypertension, Insulin resistance

## Abstract

We previously mapped hypertension-related insulin resistance quantitative trait loci (QTLs) to rat chromosomes 4, 12 and 16 using adipocytes from F2 crosses between spontaneously hypertensive (SHR) and Wistar Kyoto (WKY) rats, and subsequently identified *Cd36* as the gene underlying the chromosome 4 locus. The identity of the chromosome 12 and 16 genes remains unknown. To identify whole-body phenotypes associated with the chromosome 12 and 16 linkage regions, we generated and characterised new congenic strains, with WKY donor segments introgressed onto an SHR genetic background, for the chromosome 12 and 16 linkage regions. We found a >50% increase in insulin sensitivity in both the chromosome 12 and 16 strains. Blood pressure and left ventricular mass were reduced in the two congenic strains consistent with the congenic segments harbouring SHR genes for insulin resistance, hypertension and cardiac hypertrophy. Integrated genomic analysis, using physiological and whole-genome sequence data across 42 rat strains, identified variants within the congenic regions in *Upk3bl*, *RGD1565131* and *AABR06087018.1* that were associated with blood pressure, cardiac mass and insulin sensitivity. Quantitative trait transcript analysis across 29 recombinant inbred strains showed correlation between expression of *Hspb1*, *Zkscan5* and *Pdgfrl* with adipocyte volume, systolic blood pressure and cardiac mass, respectively. Comparative genome analysis showed a marked enrichment of orthologues for human GWAS-associated genes for insulin resistance within the syntenic regions of both the chromosome 12 and 16 congenic intervals. Our study defines whole-body phenotypes associated with the SHR chromosome 12 and 16 insulin-resistance QTLs, identifies candidate genes for these SHR QTLs and finds human orthologues of rat genes in these regions that associate with related human traits. Further study of these genes in the congenic strains will lead to robust identification of the underlying genes and cellular mechanisms.

## Abstract (precis)

**Summary:** Comparative genome analyses identify candidate genes for hypertension and insulin resistance on rat chromosomes 12 and 16, and marked enrichment of insulin resistance genes in the syntenic regions of the human genome.

## INTRODUCTION

High blood pressure and type 2 diabetes affect over 1 billion people worldwide and the two conditions frequently co-exist (Shaw et al., 2010; Vos et al., 2015). Although successful blood pressure treatment can reduce stroke risk by up to 40%, concomitant reductions in myocardial infarction are less pronounced (15-25%), suggesting a role of insulin resistance and other metabolic defects in susceptibility to myocardial infarction (Wright et al., 2015). Large numbers of loci associated with hypertension and insulin resistance have been identified through human genome-wide association studies (GWAS). However, the molecular mechanisms underlying most of these associations remain elusive, with many residing in non-coding regions of the genome, having small gene effects and carrying associations across multiple adjacent genes (Manolio et al., 2009).

Genetic studies in animal models provide important opportunities for identifying the genes and mechanisms underlying disease traits. Experimental crosses in rats and mice, including mapping studies in congenic strains, have been used to identify hundreds of physiological and pathophysiological quantitative trait loci (QTLs) for complex traits such as blood pressure, left ventricular (LV) mass and type 2 diabetes (Aitman et al., 2008). Whilst genes underlying these QTLs have in some cases been identified and translated to mice and humans (Aitman et al., 2008; McDermott-Roe et al., 2011; Nabika et al., 2012; Petretto et al., 2008), the overwhelming majority of rodent QTL genes remain unidentified.

The spontaneously hypertensive rat (SHR), the most widely studied model of human essential hypertension, also manifests insulin resistance and LV hypertrophy – traits that commonly coexist with human hypertension (DeFronzo, 1988; Ferrannini et al., 1987; Lusis et al., 2008). Using crosses between SHR and Wistar Kyoto (WKY) rats, we identified three QTLs on chromosomes 4, 12 and 16 linked to adipose insulin sensitivity (Aitman et al., 1997). Further exploration of the chromosome 4 linkage by expression analysis and congenic mapping identified *Cd36* as the major determinant of SHR hypertension and insulin resistance in this chromosomal region, a result followed by the demonstration of associations between *CD36*, hypertension and related metabolic traits in mice and humans (Aitman et al., 1999; Corpeleijn et al., 2006; Farook et al., 2012; Love-Gregory et al., 2008, 2011; Pietka et al., 2014; Wilson et al., 2016).

The aims of the present study were to pursue the linkages to hypertension and insulin-related metabolic traits on SHR chromosomes 12 and 16 by generating and characterising new SHR congenic lines and testing candidate genes by analysis of gene expression and *in silico* comparative genomic analysis across 42 rat strains, and between rats and humans. We demonstrate strong linkage between blood pressure, LV mass, *in vivo* insulin action and the congenic regions of SHR chromosomes 12 and 16, and show significant enrichment for genes associated in human GWAS with insulin action in the regions of the human genome that are syntenic to these rat congenic regions.

## RESULTS

### Body mass and energy homeostasis

**Fig. 1.**
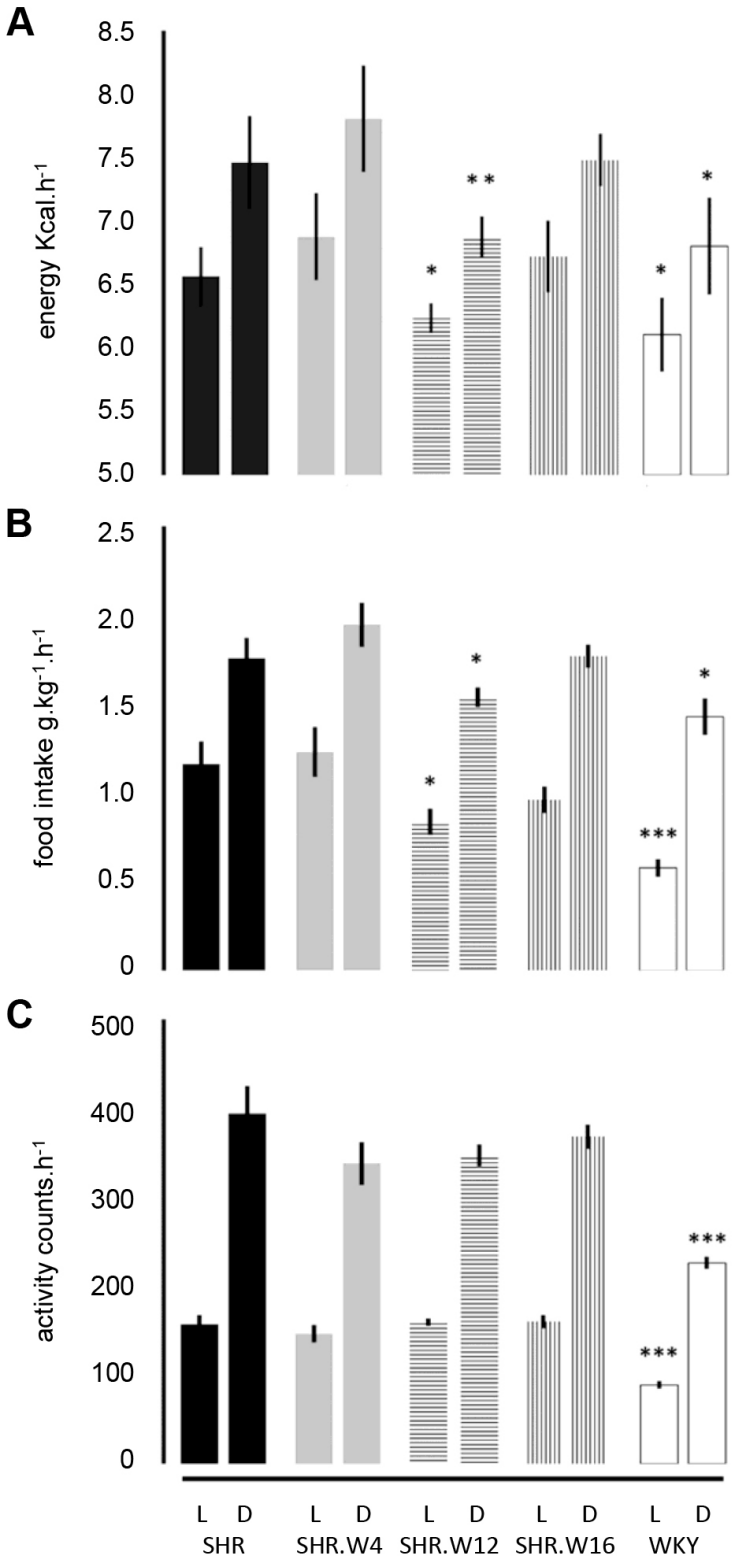
**Indirect calorimetry, activity and food intake.** (A) Energy expenditure, (B) food intake and (C) activity at age 15 weeks in parental SHR and WKY and congenic strains (*n*=10-16 rats per strain). Bar and whiskers show mean±95% CI; *P<0.05, **P<0.005, ***P<0.0001, compared with SHR. L, light; D, dark.

Body masses across congenic strains were similar, except for SHR.W16, which, on average, weighed 14 g less than SHR (Table 1). WKY rats had heavier epididymal and retroperitoneal fat pads compared with SHR (Table 1). Both SHR.W4 and SHR.W12 had similar epididymal, but heavier retroperitoneal fat pads than SHR (Table 1). Differences in expended energy, food intake and activity were found among the various strains. Compared with SHR, WKY rats expended less energy diurnally and nocturnally, consumed less food and had lower activity counts (Fig. 1A-C; Fig. S1A-C). SHR.W12 energy expenditure and food intake was lower than in SHR (Fig. 1A,B; Fig S1A,B), while the other congenics had similar energy expenditure and food intake to SHR. Circulating leptin levels were similar across strains (SHR, 2.12±0.32 ng ml^−1^; SHR.W4, 3.19±0.51 ng ml^−1^; SHR.W12, 1.72±0.26 ng ml^−1^; SHR.W16, 1.80±0.40 ng ml^−1^; WKY, 1.89±0.25 ng ml^−1^; *P*>0.05, one-way ANOVA followed by Fisher's LSD test).

**Table 1.**
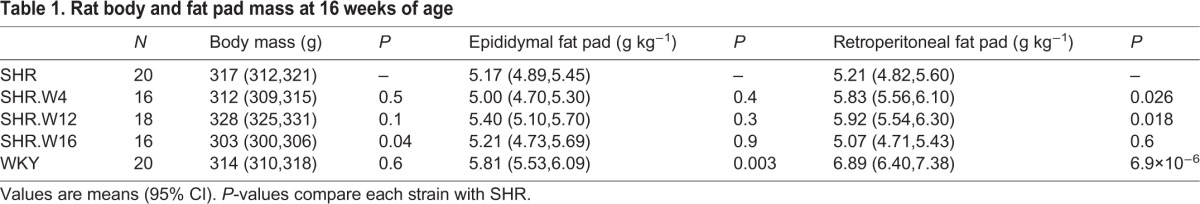
**Rat body and fat pad mass at 16 weeks of age**

### *In vivo* insulin sensitivity

**Fig. 2.**
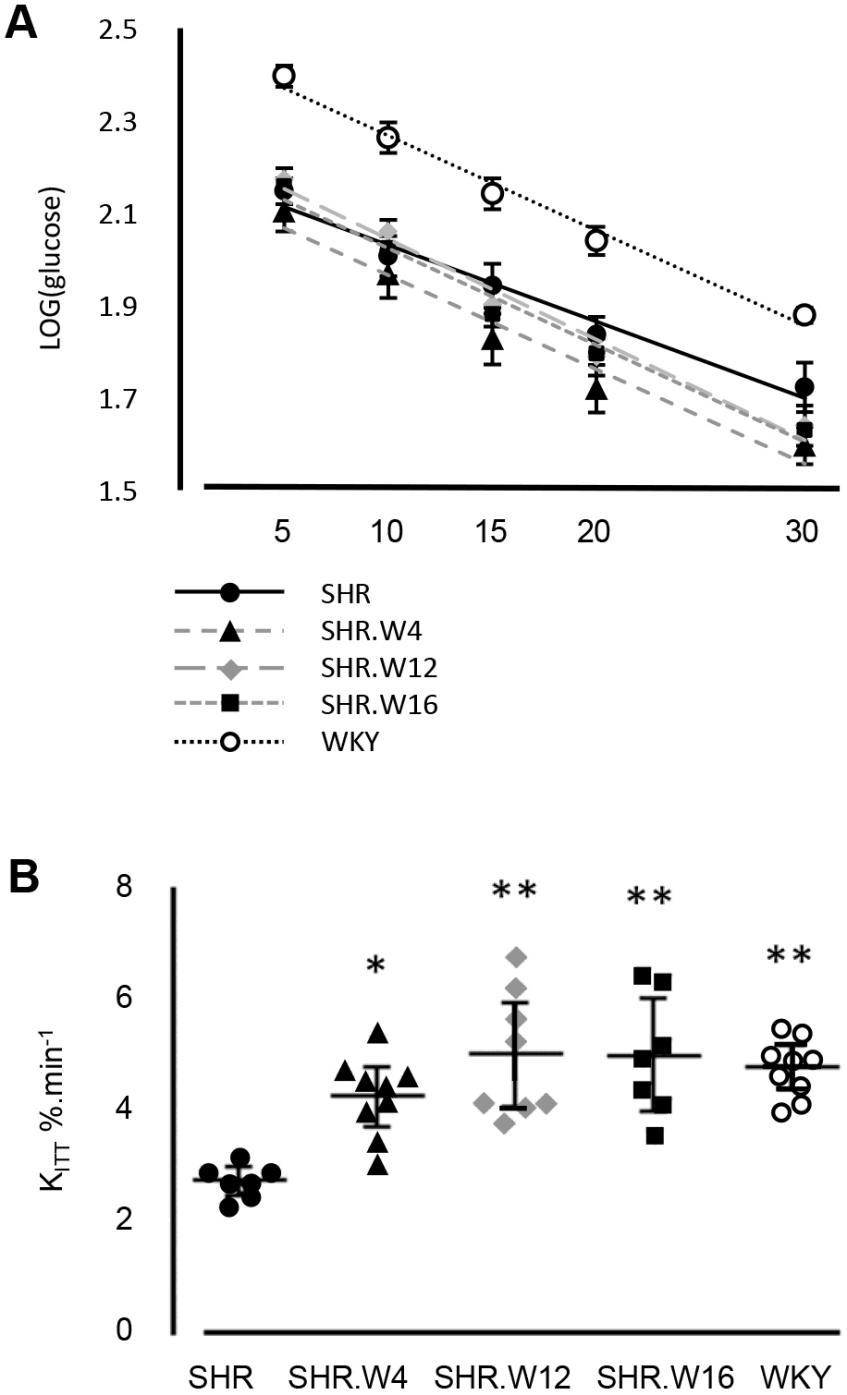
***In vivo* insulin-mediated glucose clearance.** (A) Log(glucose) disappearance 5-30 min after insulin bolus and (B) insulin-stimulated plasma glucose clearance (*K*_ITT_) in parental and congenic strains (*n*=7-10 rats per strain). Mean±95% CI; *P=0.0008, **P<0.0001, compared with SHR.

We determined insulin-stimulated glucose clearance (*K*_ITT_) to assess each strain's ability to remove blood glucose in response to insulin (AUC_insulin_: SHR, 990±145; SHR.W4, 1144±110; SHR.W12, 1106±92; SHR.W16, 1078±82; WKY, 1092±89; *P*>0.05) (Fig. 2A,B). WKY *K*_ITT_ was 2.04% min^−1^ greater than SHR (Fig. 2B). *K*_ITT_ in the congenic rats was also significantly augmented: SHR.W4 cleared glucose at 1.50% min^−1^, SHR.W12 at 2.27% min^−1^ and SHR.W16 at 2.25% min^−1^, faster than SHR (Fig. 2B).

### Hepatic and skeletal muscle triglycerides

The triglyceride content of the liver was not significantly different across parental and congenic strains, although WKY and SHR.W16 tended to be lower than SHR, SHR.W4 and SHR.W12 (SHR, 1.22±0.31 mM; SHR.W4, 0.96±0.37 mM; SHR.W12, 0.82±0.28 mM; SHR.W16, 0.54±0.26 mM; WKY, 0.49±0.23 mM; *P*>0.05). We found no significant differences in skeletal muscle triglyceride content; although SHR.W4 and SHR.W12 tended towards WKY levels (SHR, 0.37±0.4 mM; SHR.W4, 0.99±0.39 mM; SHR.W12, 0.86±0.67 mM; SHR.W16, 0.54±0.28 mM; WKY, 1.18±0.79 mM; *P*>0.05).

### Hypertension and cardiac hypertrophy

**Fig. 3.**
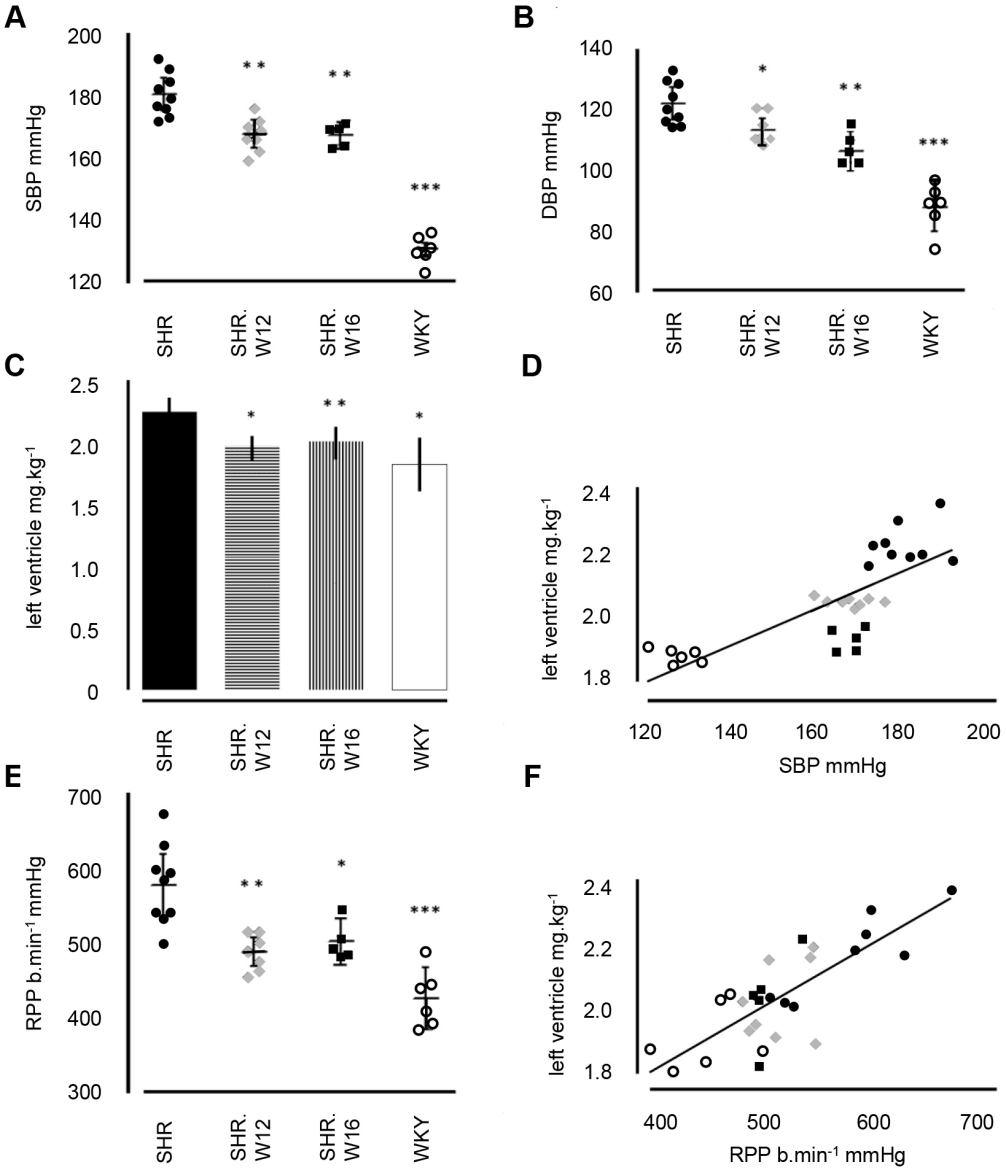
**Blood pressure, heart and left ventricular mass.** (A) Systolic (SBP), (B) diastolic blood pressure (DBP), (C) relative left ventricle (LV) mass, (D) scatter plot showing relationship between SBP and LV mass across strains, (E) rate pressure product (RPP), (F) scatter plot showing correlation between RPP and LV weight across strains. *P<0.05, **P<0.005, ***P<0.0001, between SHR and WKY/congenic strains (*n*=5-9 rats per strain). *P*>0.05, differences in left ventricle mass between WKY and congenic strains. Mean±95% CI.

We showed previously that hypertension in SHR, linked to chromosome 4 in recombinant inbred and congenic strains, is, in part, caused by renal Cd36 deficiency, as a component of the overall metabolic syndrome abnormalities encoded by SHR *Cd36* (Aitman et al., 1999; Neckar et al., 2012; Pravenec et al., 1999, 2008). Therefore, we decided to investigate blood pressure (BP) and cardiac hypertrophy in the chromosome 12 and 16 lines. The SHR had significantly higher mean BP (182/122 mmHg) than WKY (129/88 mmHg) (Fig. 3A,B). Both SHR.W12 and SHR.W16 had significantly lower BP than SHR (172/115 and 172/110 mmHg, respectively, in SHR.W12 and SHR.W16; Fig. 3A,B).

Relative LV mass in WKY, SHR.W12 and SHR.W16 (mean values 1.83, 2.02 and 1.90 g kg^−1^, respectively) was significantly lower than in SHR (2.21 g kg^−1^; Fig. 3C). A significant positive relationship was found between systolic BP and LV mass (*r*^2^=0.618, *P*<0.0001; Fig. 3D). Relative heart mass was similar across strains (mean values: SHR, 3.98; SHR.W12, 3.84; SHR.W16, 4.08; WKY, 3.89 g kg^−1^; all *P>*0.05).

Heart rate varied between strains, with WKY having a significantly higher (+16 bpm) and SHR.W12 significantly lower (−20 bpm) heart rate than SHR (Table 2). Heart rate combined with systolic blood pressure, the rate pressure product (RPP), was lower (−147 bpm mmHg) in WKY than SHR (Fig. 3E). Both congenic strains had reduced RPP that was 89 and 75 bpm mmHg lower than SHR (Fig. 3E). RPP was highly correlated with LV mass (*r^2^*=0.621, *P*<0.0001; Fig. 3F).

**Table 2.**
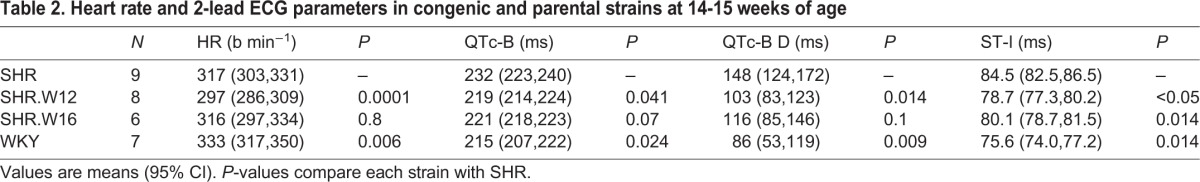
**Heart rate and 2-lead ECG parameters in congenic and parental strains at 14-15 weeks of age**

We used 2-lead ECG to detect alterations in ventricular polarisation related to cardiac hypertrophy. WKY had significantly shorter corrected QT interval (QTc-B), QT dispersion (QTD) and ST interval (ST-I) than SHR (Table 2). SHR.W12 QTc-B (−13 ms), QTD (−45 ms) and ST-I (−4.4 ms) were all shorter than SHR; ST-I alone was shorter (−5.8 ms) in SHR.W16 than SHR (*P*<0.05).

### Expression analysis of genes in the chromosome 12 and 16 loci

Thirty-two genes within the chromosome 12 congenic region and 32 genes in the chromosome 16 congenic region were found from previous expression quantitative trait locus (eQTL) and quantitative trait transcript (QTT) data (Langley et al., 2013; Morrissey et al., 2011) to be either *cis*-eQTLs or to have gene expression that correlated across the BXH/HXB recombinant inbred strain panel, in one or more tissues, with hypertension or insulin resistance-related traits. We selected 14 of these 64 genes that showed a QTT correlation in adrenal, kidney or left ventricle, related to hypertension; or showed a QTT correlation in fat, liver or skeletal muscle related to whole-body glucose homeostasis or tissue-specific metabolic phenotypes, for differential expression analysis in the relevant tissue, between the SHR, WKY and the chromosome 12 and 16 congenic strains (data not shown). Four genes – three on chromosome 12 and one on chromosome 16 – showed differential expression between SHR and WKY, and in addition showed similar expression between the relevant congenic strain and WKY, indicating *cis*-regulated control of gene expression from within the congenic segment (Table 3).

**Table 3.**
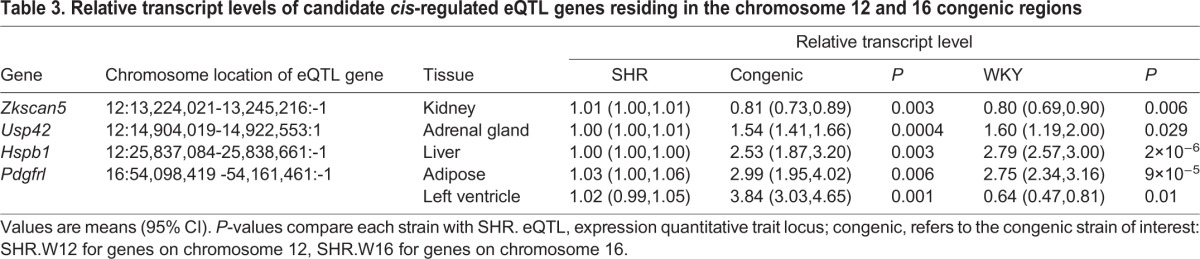
**Relative transcript levels of candidate *cis*-regulated eQTL genes residing in the chromosome 12 and 16 congenic regions**

*Zkscan5* (zinc finger with KRAB and SCAN domains 5), a transcript inversely correlated with systolic blood pressure in the kidney, was 1.25-fold lower in abundance in kidney tissue in SHR.W12 and WKY compared with SHR (Table 3, Table S4). Two transcripts, adrenal ubiquitin-specific peptidase 42 (*Usp42*) and hepatic heat shock protein β1 *Hspb1* (inversely correlated to adipocyte volume), were elevated in SHR.W12 and WKY (1.57- and 2.7-fold on average, respectively) compared with SHR (Table 3, Table S4).

On chromosome 16, platelet-derived growth factor receptor-like (*Pdgfrl*), which correlates with ‘delta captopril effects’ in the left ventricle, was differently expressed in SHR.W16 and WKY compared with SHR; WKY expression was reduced, whereas in SHR.W16, expression was increased. However, expression of *Pdgfrl* in adipose tissue was more closely matched in SHR.W16 to WKY (2.4- and 3.3-fold, respectively) than to SHR (Table 3, Table S4).

### Loci on chromosomes 12 and 16 harbour variants related to insulin resistance, hypertension and hypertrophy

In order to identify deleterious single-nucleotide variants (SNVs) present in the SHR congenic regions on chromosomes 12 and 16 (and absent in WKY), that were associated with insulin resistance, hypertension and LV hypertrophy, we used the PLINK tool (Purcell, 2014) to integrate relevant phenotype data with SNV data from 42 rat strains. We located all SNVs together with their PLINK-assigned *P*-values (indicating closeness of SNV-to-phenotype relationship), for those SNVs with *P*<0.01 (Fig. S2).

Missense variants in the coding regions of 12 genes with *P*<1×10^−4^ were considered most likely to affect the phenotypes of interest in SHR, causing a predicted deleterious amino acid substitution (Table 4). SNVs in *Dtx2*, *Upk3b* and *Upk3bl* were found to be significantly associated with both insulin resistance and hypertension (Table 4). Five SNVs in genes *AABR06087018.1*, *Pms2*, *Zfp866*, *Gatad2a* and *Daglb* were found to be exclusively associated with insulin resistance. In addition, two SNVs in genes *RGD1565131* and *Grid2ip* were found to be associated with cardiac hypertrophy (Table 4).

**Table 4.**
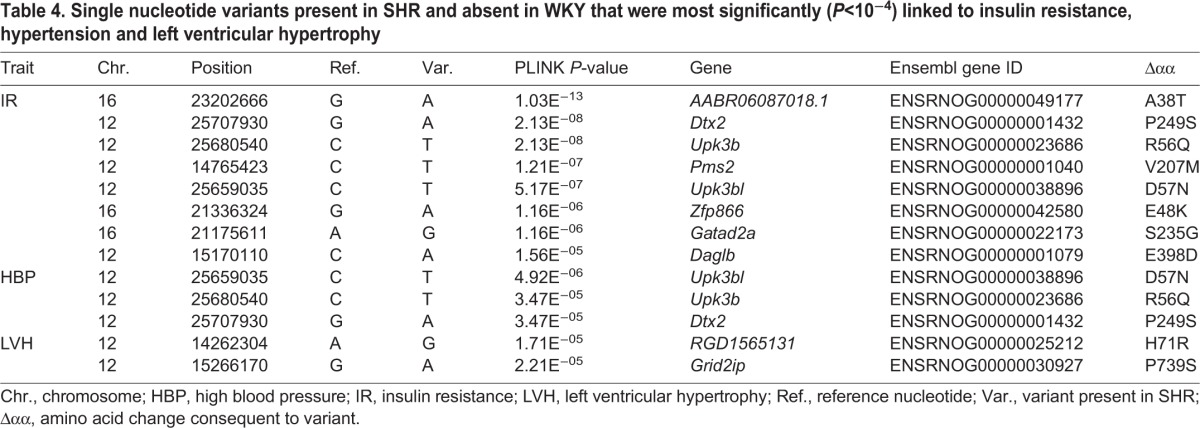
**Single nucleotide variants present in SHR and absent in WKY that were most significantly (*P*<10^−4^) linked to insulin resistance, hypertension and left ventricular hypertrophy**

### Identification of human GWAS hits within the syntenic regions of the donor congenic segments

To investigate whether the chromosomal segments of the human genome that are syntenic to rat chromosome 12 and 16 congenic regions contain genes identified as human GWAS hits for relevant cardio-metabolic traits (including hypertension, left ventricular hypertrophy, insulin resistance and type 2 diabetes) we mined the syntenic regions in the human genome for reported GWAS hits (Table S1).

We identified GWAS hits associated with type 2 diabetes, metabolic syndrome and insulin resistance-related traits in five genes in the human synteny regions of the chromosome 12 congenic strain, and with 10 genes in the synteny regions of the chromosome 16 congenic strain (Table 5, Table S5). This represents a significant enrichment for GWAS hits in these chromosomal regions (chromosome 12 synteny regions, *P*<0.003; chromosome 16 synteny region, *P*<6.7×10^−13^; Table 5).

**Table 5.**
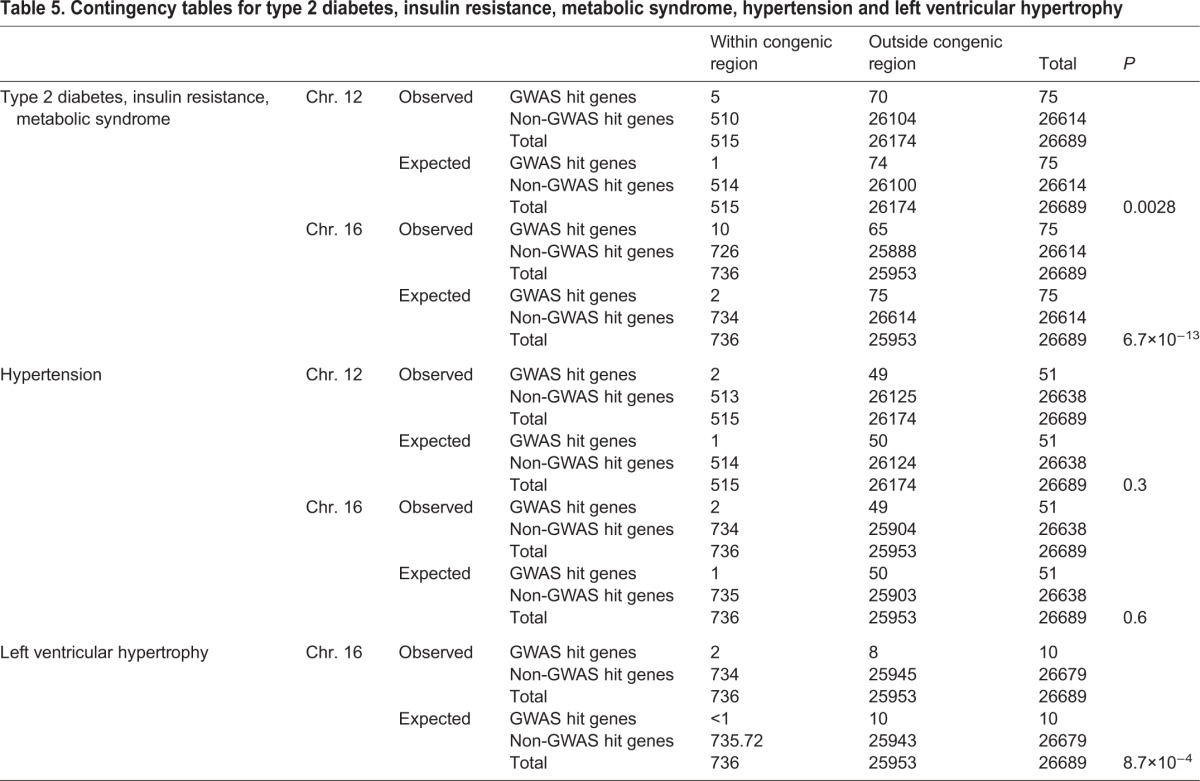
**Contingency tables for type 2 diabetes, insulin resistance, metabolic syndrome, hypertension and left ventricular hypertrophy**

GWAS hits associated with blood pressure were identified in two genes in the chromosome 12 congenic strain synteny regions and two genes in the chromosome 16 congenic strain. GWAS hits for LV mass were not identified in the chromosome 12 synteny regions but were identified in two genes in the chromosome 16 synteny region, one of these also being associated with blood pressure. Syntenic segments of neither region were significantly enriched for genes or GWAS hits for blood pressure-related traits, but the syntenic regions of the chromosome 16 region were significantly enriched for GWAS hit genes for left ventricular (LV) mass (*P*<8.7×10^−4^) (Table 5). Three genes, *SLC12A9* (chromosome 12), *CACNA1D* and *CSMD1* (chromosome 16), were associated with both blood pressure and insulin resistance-related metabolic traits (Table S5).

## DISCUSSION

Hypertension and insulin resistance frequently co-occur in the same individual. Pathologically increased blood pressure leads to increased myocardial load, while insulin resistance affects myocardial metabolism and vascular reactivity, leading to compensatory LV hypertrophy and failure (Evrengul et al., 2005; Kaftan et al., 2006; Roman and Devereux, 2014).

We previously mapped QTLs on SHR chromosomes 4, 12 and 16 that were associated with reduced insulin-stimulated glucose uptake in isolated adipocytes from hypertensive SHR compared with normotensive WKY (Aitman et al., 1997). Subsequent studies investigating the chromosome 4 region identified *Cd36*, a fatty acid translocase, as the gene underlying both insulin sensitivity and blood pressure QTLs on SHR chromosome 4 (Aitman et al., 1999; Pravenec et al., 2008). *CD36* has since been found to be important in the development of type 2 diabetes and essential hypertension in humans (Corpeleijn et al., 2006; Lepretre et al., 2004; Wang et al., 2012).

In this study, we explored regions on SHR chromosomes 12 and 16 and their function in regulating blood pressure, cardiac hypertrophy and insulin resistance. Cardiovascular and metabolic phenotyping of the congenic strains showed that the chromosome 12 and 16 congenic strains had captured important genes for both cardiovascular and metabolic phenotypes within the congenic segments. Systolic blood pressure was reduced by around 10 mmHg in both of the congenic strains, equivalent to ∼20% of the difference between SHR and WKY. The variation in systolic blood pressure across the SHR, WKY, and two congenic strains explained over 60% of the variation in both left ventricular mass and left ventricular load across the four strains (Fig. 3E,F). The reduction in left ventricular mass and myocardial load in either of the two congenic strains compared with parental SHR rats was almost equivalent to the total difference between the SHR and WKY strains. In metabolic terms, insulin tolerance testing showed an increase in *K*_ITT_, the glucose disposal rate, by over 50% in both congenic strains compared with the insulin-resistant SHR parental strain. As with several of the cardiovascular traits, this increase in insulin action was similar to the entire difference between the SHR and WKY parental lines.

We combined physiological, eQTL and differential expression data to identify candidate genes for SHR insulin resistance and blood pressure encoded on these congenic segments. Of the 64 genes within the chromosome 12 and 16 congenic intervals that were identified previously as either *cis*-eQTL genes in relevant tissues or correlated in QTT analysis with relevant cardio-metabolic phenotypes, nine genes on chromosome 12 and five genes on chromosome 16 were tested because of correlation in QTT, with the most plausible tissue expressing the differentially expressed gene. Three genes from chromosome 12 – *Hspb1*, *Zkscan5* and *Usp42* – were found to be differentially expressed between SHR and the congenic strain in the tissue of interest. Of the proteins encoded by these genes, Hspb1 (also known as Hsp27) has been the most studied and is involved in numerous cellular processes, including protection from oxidative stress (Acunzo et al., 2012; Matsumoto et al., 2015). This metabolic stress protein has been found to improve insulin signalling when induced in monocytes extracted from diabetic and obese patients (Simar et al., 2012). *Hsp27* was also upregulated with *Pparg* and *Hsp72* by the experimental compound naringin, which improves insulin sensitivity and lipid metabolism in rats (Sharma et al., 2011). Scant information is available for Zkscan5, although one GWAS study located a single nucleotide polymorphism (SNP) with significant association to dehydroepiandrosterone sulfate levels, which have been linked to pulmonary hypertension (Ventetuolo et al., 2016; Zhai et al., 2011). Ubiquitin-specific peptidase 42 (Usp42), stabilises p53 in response to stress and is a transcriptional regulator; however, there is currently no study linking the gene to cardio-metabolic traits (Hock et al., 2014).

Of the five genes on chromosome 16 that were correlated in QTT with cardio-metabolic phenotypes, only one gene, *Pdgfrl*, was found to be differentially expressed between SHR and congenic strain adipose tissue, showing similarly increased expression in SHR.W16 and WKY compared with SHR, in epididymal fat. The PDGF signalling pathway regulates a number of processes, including angiogenesis, wound healing and inflammation (Hu and Huang, 2015). Moreover, low levels of PDGF-BB, a component of this signalling pathway, is linked to cardiovascular events in type 2 diabetics (Yeboah et al., 2007).

From linkage disequilibrium analysis across 42 rat strains, we found SHR SNVs in 10 genes in the chromosome 12 and 16 congenic regions that were significantly associated across the 42 rat strains with hypertension, hypertrophy and insulin resistance in the SHR. Three genes within the chromosome 12 congenic strain interval (*Dtx2*, *Upk3b*, *Upk3bl*) contain SNVs that were significantly associated across 42 rat strains with both hypertension and insulin resistance. Deltex 2 E3 ubiquitin ligase (Dtx2), acts as a negative regulator of Notch signals in mature T-cells (Lehar and Bevan, 2006). Inhibition of Notch signalling has been reported to improve insulin resistance (Pajvani et al., 2011). Furthermore, *Notch2* SNPs have been reported in a number of GWAS studies associated with cardiovascular disease and type 2 diabetes (Qi et al., 2013; Zeggini et al., 2008). Therefore, it is plausible that the SHR variant of *Dtx2* has a reduced ability to negatively regulate Notch signals in relation to downstream insulin signalling, although this will require testing in future studies.

Uroplakin 3b (Upk3b), is a minor component in the asymmetric unit membrane of the urothelium (Yu et al., 1994) and *Upk3b* expression in the early mouse embryo is reportedly restricted to mesothelial cells and epicardium (Huang et al., 2012). However, little is known about the physiological function of *Upk3b* and no association with hypertension or insulin action has been reported hitherto. The related gene *Upk3bl* has scant published information regarding its function, but may have involvement in the rare disease rhabdoid glioblastoma (Koh et al., 2015).

Our human-rat comparative analysis of the chromosome 12 and 16 congenic segments indicated a significant enrichment for insulin resistance-related GWAS hit genes on the regions syntenic to both congenic segments, with 5 and 10 GWAS hit genes for these traits within the syntenic regions to the chromosome 12 and 16 congenic segments, respectively. We also found an enrichment, although less significant, for GWAS hit genes for cardiac hypertrophy in the regions syntenic to the chromosome 16 congenic strain.

Three of the GWAS genes (*SLC2A9*, *CACNA1D* and *CSMD1*), one in the chromosome 12 and two in the chromosome 16 synteny groups, harboured SNPs significantly associated with both blood pressure and insulin resistance/type 2 diabetes in humans. *SLC2A9* encodes an electroneutral inorganic cation-chloride co-transporter whose function has yet to be fully established (Gagnon and Delpire, 2013). *CACNA1D* encodes a voltage-dependent calcium channel, and SNPs in this gene are associated by GWAS with insulin resistance in African Americans, and with blood pressure in Chinese and people of African ancestry (Irvin et al., 2011; Lu et al., 2015; Zhu et al., 2015). Deletion of *Cacna1d* in mice was expected to affect insulin secretion and result in hyperglycaemia; however, this was not observed (Platzer et al., 2000). However, the authors did find that *Cacna1d^−/−^* mice were affected by arrhythmia and reduced heart rate compared with the wild type (Platzer et al., 2000).

*CSMD1* encodes the cub and sushi domains 1 protein and, as for *CACNA1D*, SNPs in this gene are associated with insulin resistance in African Americans, and with blood pressure in the Han Chinese population (He et al., 2013; Irvin et al., 2011). Functionally, Csmd has been found to negatively regulate the classical complement pathway (Kraus et al., 2006). Thus, given the growing evidence of the role of complement in cardiovascular and metabolic disease (Hertle et al., 2014), this gene may be important in preventing over-activity of the complement system – a phenomenon observed in both cardiovascular and metabolic disease.

The glutamate receptor ionotropic delta 1 gene *GRID1* was one of two GWAS orthologues for LV hypertrophy in the chromosome 16 congenic region (Vasan et al., 2009). *Grid1^−/−^* mice were not reported to have a cardiovascular phenotype (Gao et al., 2010); however, deletion of *GRID1* and surrounding genes in humans, was associated with cardiac defects (van Bon et al., 2011). The second hypertrophy-related gene neuregulin 3 (*NRG3*) has unknown function; however, one study in the rat showed downregulation of neuregulin receptor signalling in the transition from hypertrophy to failure in rats (Rohrbach et al., 1999).

Our study provides a complementary view to a recently published study by Sedova et al. (2016), which carried out blood pressure and metabolic testing and an appraisal of SNVs on a single SHR congenic strain for chromosome 16. Although these two studies highlight some genes in common (such as the plausible candidates *Lpl* and *Gatad2a*), our study, of congenic strains on chromosomes 12 and 16, was carried out in linkage regions to adipocyte insulin resistance that we had defined previously. Sedova carried out a limited appraisal of SNVs between the two parental strains in the chromosome 16 congenic region. Our study may have more plausibility, as it drew upon extensive gene expression and QTT data, as well as linkage disequilibrium data across 42 rat strains, to filter and select candidate genes within the congenic regions.

The genes and mechanisms underlying hypertension and insulin resistance, in humans and rats, are yet to be fully elucidated. Here, we combined genetic, physiological and comparative genomic analyses to define chromosomal regions underlying SHR blood pressure, insulin sensitivity and left ventricular mass, identifying candidate genes and finding human orthologues of rat genes in these regions that associate with these traits. Further study of these genes in the congenic strains will lead to robust identification of the underlying genes and cellular mechanisms.

## MATERIALS AND METHODS

### Generation of congenic strains

Congenic lines containing regions of WKY chromosomes 12 and 16 on an SHR background were constructed with a speed congenic approach using SHR/NCrl as recipient genome and WKY/NCrl (Charles River Laboratories, Margate, UK) as donor genome (Markel et al., 1997). A chromosome 4 congenic line that captured *Cd36* was generated for comparative purposes. Microsatellite marker analysis was carried out at each backcross (Tables S2 and S3), and progeny heterozygous for relevant congenic segments with the lowest proportion of WKY background genotypes were selected as breeders. Once all background microsatellites were confirmed as homozygous SHR, each of the chromosome 4, 12 and 16 lines were intercrossed. Offspring that were homozygous for WKY in the congenic interval (using microsatellites in Table S3) were brother-sister mated to fix the congenic interval. The lines were designated SHR.W4-(D4rat143-D4rat10)/Tja (SHR.W4), containing a congenic segment of 24.2 Mb; SHR.W12-(D12rat1-D12mit3)/Tja (SHR.W12), containing a congenic segment of 28.6 Mb; and SHR.W16-(D16rat88-D16rat15)/Tja (SHR.W16), containing a congenic segment of 79.2 Mb. All animals were maintained under 12 h:12 h light-dark cycle with free access to food and water. Experimental procedures were approved by the UK Home Office under the Animals (Scientific) Procedures Act 1986.

### Energy expenditure, food intake and activity

Indirect calorimetry, food intake and activity were assessed using the Oxymax Lab Animal Monitoring System (CLAMS, Columbus Instruments, Columbus, OH, USA) for 72 h (24 h acclimatisation; 48 h data collection) with free access to water and chow. Energy expenditure was calculated according to McLean and Tobin (CV, calorific value, kcal kg^−1^ h^−1^) (McLean and Tobin, 1987). Serum leptin (*n*=6 per group) was measured by ELISA (Millipore, UK).

### Whole-body insulin sensitivity

Insulin-stimulated glucose clearance (Actrapid; Novo Nordisk, Bagsvaerd, Denmark) was measured by glucometer (Contour, Bayer Healthcare, Basel, Switzerland), in venous blood drawn from the tail vein of overnight-fasted rats with free access to water, 5-30 min following an intravenous insulin bolus, according to Eskens et al. (2013), and using an insulin dose, 1 U kg^−1^, previously used to reliably assess whole-body response to insulin (Diaz-Castroverde S, 2016; Lambert et al., 2016; Moak et al., 2014). Rats were anaesthetised throughout in order to reduce stress associated with hypoglycaemia and this has not been found to affect the interpretation of insulin sensitivity in the same rat strains (Hulman et al., 1991, 1993). Body temperature was controlled using the Homeothermic Monitoring System (Harvard Apparatus). Plasma insulin was determined by ELISA (Rat/Mouse Insulin ELISA, Millipore). Glucose clearance (*K*_ITT,_ % min^−1^) was calculated from the log(glucose) disappearance curve 5-30 min (Fig. 2A) as 0.693/*t*_1/2_×100 (Lundbaek, 1962).

### Hepatic and skeletal muscle triglyceride analysis

Lipid was extracted from liver and soleus muscle (*n*=4 per group) according to manufacturer's instructions and analysed using the Triglyceride Quantification Kit (Abcam, Cambridge, UK).

### Blood pressure and ECG telemetry

Blood pressure and 2-lead ECG were measured in conscious, free-moving 14- to 15-week-old male rats. A blood pressure ECG radio-telemetry device was surgically implanted in accordance with the manufacturer's instructions and cardiovascular measurements taken at least 1 week after implantation (HD-S11; Data Sciences International, Roermond, The Netherlands). Blood pressure, heart rate and QT interval were measured for 5 min h^−1^ over a consecutive 48 h period, analysed using Ponemah (Ponemah Data Analysis v5.20, Data Sciences International, Roermond, The Netherlands) and presented as mean values over this time period. Rate pressure product was calculated as systolic blood pressure×heart rate×10^−2^ (Gobel et al., 1978). Bazett's correction for QT was calculated as QTc-B=QT/(RR)^1/2^ (Kmecova and Klimas, 2010); QTc-B D was calculated as the difference between maximum and minimum QTc-B (de Bruyne et al., 1998).

### Expression and variant analyses of genes in the chromosomes 12 and 16 QTLs

We used two approaches to identify candidate genes for the SHR traits investigated in this study: first, we used previously generated eQTL (Langley et al., 2013; Petretto et al., 2006) and quantitative trait transcript (QTT) (Morrissey et al., 2011) data to identify eQTL genes within the congenic intervals and genes that correlated with (patho)physiological traits across the BXH/HXB recombinant inbred strain panel. Relative transcript levels were quantified by real-time quantitative PCR (qPCR) from cDNA reverse transcribed from total RNA of 4-5 animals per group (iScript cDNA Synthesis Kit, Bio-Rad) extracted with Tri-reagent (Sigma) from adrenal gland, epididymal fat, kidney, left ventricle, liver and skeletal muscle. Primers were designed using Primer-BLAST software (http://www.ncbi.nlm.nih.gov/tools/primer-blast/) with sequences verified for absence of single nucleotide polymorphisms using SHR and WKY whole-genome sequences and the Integrated Genomics Viewer (http://www.broadinstitute.org/igv/). qPCR was carried out on the 7900HT Fast Real-Time PCR System (Thermo Fisher Scientific). *C*_T_ values were analysed using the 2^−ΔΔCT^ method normalising to β-actin gene, with SHR used to compare strains.

Second, we used PLINK to rank SHR SNVs within the congenic intervals on chromosome 12 and 16, with the closest relationship to each trait of interest (insulin resistance, hypertension, hypertrophy), allowing us to compare deleterious SNVs present in SHR and absent in WKY (Atanur et al., 2013; Purcell, 2014). We obtained metabolic and cardiovascular data from the Rat Genome Database on 42 rat strains and grouped strains depending on glucose and insulin levels, blood pressure, and left ventricular mass (http://rgd.mcw.edu/wg/phenotype-data13). We then generated .map and .ped input files for the PLINK program from a .vcf file containing all SNVs of the 42 strains (Hermsen et al., 2015). The original genotype calls were adjusted such that heterozygous calls were made if >25% and <75% of the reads supported a non-reference genotype, otherwise they were called as homozygous. The effects of SNVs on genes, transcripts and protein sequence were evaluated using a locally installed Ensembl Variant Effect Predictor (VEP) (http://www.ensembl.org/info/docs/tools/vep/script/index.html).

The VEP analysis was based on rat genome assembly Rn0r_5.0 and Ensembl release 78 annotations. The PLINK analysis was performed as standard case/control association analysis. We used the additional PLINK options ‘–assoc’ and ‘−allow-no-sex’.

### Comparative analysis of the congenic intervals with corresponding regions in the human genome

Using the Virtual Comparative Map (VCMap), we located regions of the human genome orthologous to the chromosome 12 and 16 congenic intervals (Kwitek et al., 2001). With the genomic coordinates corresponding to the congenic intervals, we identified human SNPs associated with ‘non-insulin-dependent diabetes mellitus’ and ‘susceptibility to essential hypertension’ using Ensembl's BioMart (Cunningham et al., 2015). In addition, we identified SNPs in genes with genome-wide significance (including those with borderline significance, i.e. *P*=5×10^−7^) using the NHGRI-EBI GWAS catalogue (https://www.ebi.ac.uk/gwas/) connected to the following search terms: blood pressure, blood pressure (age interaction), blood pressure (anthropomorphic measures interaction), blood pressure (smoking interaction), blood pressure measurement (cold pressor test), blood pressure measurement (high sodium and potassium intervention), blood pressure measurement (high sodium intervention), blood pressure measurement (low sodium intervention), blood pressure response to hydrochlorothiazide in hypertension, blood pressure variability, cardiac hypertrophy, cardiac muscle measurement, cardiac repolarisation, cardiac structure and function, cardiovascular disease risk factors, cardiovascular heart disease in diabetics, diabetes-related insulin traits, diastolic blood pressure, diastolic blood pressure (alcohol consumption interaction), fasting glucose-related traits, fasting glucose-related traits (interaction with BMI), fasting insulin-related traits, fasting insulin-related traits (interaction with BMI), fasting plasma glucose, heart rate, hypertension, hypertension (young onset), insulin-related traits, left ventricular mass, QT interval, systolic blood pressure, systolic blood pressure (alcohol consumption interaction) and type 2 diabetes.

To test whether the congenic regions were enriched for orthologues to cardio-metabolic GWAS hits in humans, we used the above GWAS search results and information gathered from Ensembl, to establish the total number of genes within and outside the congenic regions.

### Statistical analysis

Data were analysed using Minitab 17 (Minitab Ltd, Coventry, UK). All physiological and qPCR data were analysed by one-way ANOVA followed by Fisher's least significant difference test. *P*-values in the PLINK analysis were considered significant with a nominal *P*-value <1×10^−4^. SNV and GWAS enrichment analyses were performed by chi-squared analysis. Values are expressed as mean±95% confidence intervals (CI), with values of *P*<0.05 considered significant.
